# LncRNAs, MALAT1 and lnc-DC as potential biomarkers for multiple sclerosis diagnosis

**DOI:** 10.1042/BSR20181335

**Published:** 2019-01-15

**Authors:** Olfat G. Shaker, Rania H. Mahmoud, Omayma O. Abdelaleem, Enas G. Ibrahem, Abdelrahmaan A. Mohamed, Othman M. Zaki, Noha K. Abdelghaffar, Tarek I. Ahmed, Nada F. Hemeda, Naglaa A. Ahmed, Dina F. Mansour

**Affiliations:** 1 Department of Medical Biochemistry and Molecular Biology, Faculty of Medicine, Cairo University, Cairo, Egypt; 2 Department of Medical Biochemistry and Molecular Biology, Faculty of Medicine, Fayoum University, Fayoum, Egypt; 3 Department of Medical Microbiology and Immunology, Faculty of Medicine, Fayoum University, Fayoum, Egypt; 4 Department of Clinical pathology, Faculty of Medicine, Fayoum University, Fayoum, Egypt; 5 Department of Internal medicine, Faculty of Medicine, Fayoum University, Fayoum, Egypt; 6 Department of Genetics, Faculty of Agriculture, Fayoum University, Fayoum, Egypt; 7 Department of Physiology, Faculty of Medicine, Zagazig University, Zagazig, Sharkea, Egypt; 8 Department of Neurology, Faculty of Medicine, Minia University, Minia, Egypt

**Keywords:** lncRNAs, lnc-DC, MALAT1, Multiple sclerosis

## Abstract

Long non-coding RNAs (lncRNAs) play an important role in gene regulation and show greater tissue specificity and complexity of biological functions. There is on-going research in their contribution in autoimmune diseases like multiple sclerosis (MS). Our study aimed at the evaluation of serum levels of lncRNAs, MALAT1 and lnc-DC in MS patients and the investigation of the association between these lncRNAs and the disease activity. Serum from 45 MS patients and 45 healthy controls was separated. MALAT1 and lnc-DC expression levels were assayed by qRT-PCR. MALAT1 and lnc-DC were significantly increased in MS patients (*P*=0.004 and *P*=0.006, respectively) in comparison with controls. There was a significant increase in expression of MALAT1 in secondary progressive MS (SPMS) subgroup compared with controls (*P*<0.0001); however, significant elevation of lnc-DC was demonstrated in relapsing remitting MS (RRMS) subtype (*P*=0.003) compared with normal controls. A positive association between the expression levels of MALAT1 and lnc-DC (*r* = 0.513, *P* < 0.0001) in MS patients was detected. Moreover, positive correlation was observed between MALAT1and lnc-DC in RRMS (*r* = 0.569, *P* = 0.001). Serum levels of MALAT1 and lnc-DC may serve as potential novel molecular biomarkers for MS diagnosis and may provide a new direction for its treatment.

## Introduction

Multiple sclerosis (MS) is a chronic disabling disease, causing inflammation and demyelination of central nervous system (CNS) and spinal cord, affecting people around age of 30 years specially females [1]. MS is presenting with a broad range of manifestations, such as motor impairment, visual and sensory disturbance, pain, fatigue and cognitive deficits [2]. Different theories can explain etiology of MS, such as genetic predisposition, environmental factors and recently autoimmunity. Autoreactive T cells react in an abnormal form against CNS autoantigens [3], these immune cells cross blood–brain barrier and cause inflammation, demyelination and neuroaxonal degeneration.

Many studies have aimed at identification of potential biomarkers that help in the detection of disease activity and progression. Among these biomarkers, long non-coding RNAs (lncRNAs) [4] are a class of non-coding RNAs that were discovered recently and found to be longer than 200 nucleotides in length. Bioinformatics’ approaches used to identify different types of lncRNAs [5]. Despite the function of most lncRNAs is still mostly unknown, many studies have detected the critical roles of lncRNAs in various biological processes, such as transcriptional coactivation, chromatin remodeling, post-transcriptional modification and inhibition of protein translation [6]. The lncRNAs have essential roles in the processes of embryonic development, cell differentiation and various diseases like diseases of neurodegeneration [7].

It was found that lncRNAs may have a crucial role in autoimmune diseases through activation, differentiation and imbalanced expression of immune cells (T cells, B cells, macrophages and NK cells) that have been observed in diseases of autoimmunity such as psoriasis, rheumatoid arthritis and systemic lupus erythematous (SLE) [8]. It is interesting that some lncRNAs were discovered to be dysregulated in peripheral blood mononuclear cells in MS patients suggesting their role in the pathogenesis of MS [9].

Metastasis-associated lung adenocarcinoma transcript 1 (MALAT1) is an example of lncRNAs that discovered as a prognostic marker of cancer metastasis in non-small cell lung cancer [10]. Many studies found that MALAT1 is oncogenic and have been overexpressed in several solid tumors including lung, colorectal, bladder and laryngeal cancers [11–14]. This lncRNA is expressed in numerous tissues, such as reproductive, lymphoid systems, CNS, endocrine and immune systems [15,16]. In respect to its role in nervous system, MALAT1 has regulated gene expression in neurons that involved in nuclear and synapse function and synaptogenesis [17,18].

lnc-DC is a long non-coding RNA that was found to be expressed in dendritic cells (DCs) and could mediate DCs maturation by phosphorylating transducer and activator of transcription 3 (STAT3). It was found that lnc-DC can affect the differentiation of monocytes into DCs that induce T-cell activation through its role in the transcription of downstream genes. So, lnc-DC can induce the differentiation and maturation of DCs [19].

Circulating RNAs in serum have emerged as a non-invasive diagnostic application. There is evidence demonstrating that lncRNAs are stable in human serum, so circulating cell-free lncRNAs could serve as biomarkers for many cancers [20,21]. However, serum lncRNAs MALAT1 and lnc DC expression signatures in MS patients are still unknown.

The present study aimed to investigate the role and clinical relevance of lncRNAs, MALAT1 and lnc-DC in MS disease and to examine the association between them and disease activity.

## Materials and methods

### Sample collection

In this case–control study, we collected serum samples from 45 MS patients (all cases were fulfilled the McDonald criteria for MS) [22] and 45 controls with no history of any neurological or autoimmune diseases. All patients included in our study demonstrated expanded disability status scale (EDSS) progression without evidence of relapse in the 24 months prior to collection. The MS patient group was free of MS-specific treatments (immunomodulatory therapy) within 6 months prior to collection. Controls were age and gender matched. Patients were selected from outpatient clinics and inpatient departments of Internal Medicine and Neurology, Fayoum University Hospital, Fayoum, Egypt. Patients with the following conditions were excluded from the study: (i) malignancy, (ii) severe recent infection, (iii) presence of other inflammatory or autoimmune diseases and (iv) suspected drug or alcohol abuse.

### Ethical considerations

All participants were ethnic Egyptians. The study was performed with the approval of Faculty of Medicine, Fayoum University local ethics committee and carried out in compliance with the Helsinki Declaration. Informed consent was obtained from every subject enrolled in the present study after explanation of the study.

### Blood sample processing

Blood samples were withdrawn from each subject using vacutainer system. Samples were collected in tubes with separator gels that lodge between packed cells and the top serum layer [23], permitted to clot for 15 min, and then centrifuged at 4000×***g*** for 10 min. The serum samples were separated from clotted whole blood and stored at −80°C until the time of use.

### LncRNAs MALAT1 and lnc-DC quantitation

Total RNA including non-coding RNAs was extracted from serum of MS patients by miRNeasy extraction kit (Qiagen, Valenica, CA) using QIAzollysis reagent according to the manufacturer’s instructions. Concentration of RNA was determined using NanoDrop2000 that is very accurate to measure even the small quantities of RNA (Thermo scientific, U.S.A.). Reverse transcription was carried out on extracted RNA in a final volume 20 μl reactions using RT2 first strand Kit (Qiagen, Valenica, CA) according to the manufacturer’s instructions. Gene expression levels of the studied lncRNAs were evaluated using GAPDH that is widely used as internal control for serum lncRNAs in numerous studies [20,21] according to the manufacturer’s protocol. The MALAT1 Ref Seq no (NR_002819.2) Catalog no: 330701 LPH18065A, the Lnc-DC Ref Seq Accession no (ENST00000590776.0) Catalog no: 330701 LPH38114A and the primer sequences of GAPDH were as follows: forward 5′-CCCTTCATTGACCTCAACTA-3′, reverse, 5′-TGGAAGATGGTGATGGGATT-3′. Real-time PCR was done on 20 μl reaction mixture using Rotor gene Q System (Qiagen) with the following conditions: 95°C for 10 min, followed by 45 cycles at 95°C for 15 s and 60°C for 60 s. Gene expression relative to internal control (2^−Δ*C*^_t_) was calculated. Fold change was calculated using 2^−ΔΔ*C*^_t_ for relative quantitation [24].

### Statistical analysis of data

The collected data were organized, tabulated and statistically analyzed using SPSS software statistical computer package version 18 (SPSS Inc, Chicago, U.S.A.), SAS version 9.1 and SAS Enterprise 9.4 (SAS Institute Inc, NC, U.S.A.). For quantitative data, the mean, median, standard deviation (SD) and interquartile range (IQR) were calculated. Kolmogorov–Smirnov test test was performed as a test of normality. Variables were not normally distributed, so Mann–Whitney *U*-test was used in comparing between the two groups. Qualitative data were presented as number and percentages, chi square (χ^2^) was used as a test of significance. Spearman correlation was run to identify relation of MALAT1 and Inc-DC with study parameters. Logistic regression was used to calculate probabilities and corresponding 95% CI. Receiver operating characteristic (ROC) curve was used to determine the cut-off point in which highest sensitivity and specificity of MALAT1 and Inc-DC as a predictor for MS and in differentiating secondary progressive MS (SPMS) from relapsing remitting MS (RRMS). For interpretation of results of tests of significance, significance was adopted at *P*≤0.05. Sample size was calculated using (G power version 3.0.10). Minimal sample size of patients was 45 in each group needed to get power level of 0.80, α level of 0.05 (two tailed) and medium effect size of 0.60 for (MALAT1).

## Results

### Demographic and clinical characteristics of study population

Serum samples from 45 healthy controls (consisted of 8 males and 37 females, mean age 32.4 years, SD 9.2) and 45 MS patients (consisted of 6 males and 39 females, mean 31.3 years, SD 8.3) were enrolled in the present study. Demographic and clinical characteristics of MS patients and controls are summarized in Table 1, no significant difference was observed in age and sex ratio among two groups (*P*>0.05).

**Table 1:** Distribution of study subjects according to their demographic and clinical characteristics

Variable	MS patients (*n*=45)	Healthy controls (*n*=45)	*P* value
Age/y (mean ± SD)	31.3 ± 8.3	32.4 ± 9.2	0.556^1^
Sex**:** *n* (%)			
Female	39 (86.7%)	37 (82.2%)	0.561^2^
Male	6 (13.3%)	8 (17.8%)	
MS type**:** *n* (%)			
PRMS	32 (71.1%)		
SPMS	13 (28.9%)		
**EDSS** (mean ± SD)	3.37 ± 1.94		

^1^Independent *t*-test, ^2^Chi square (χ2) test; EDSS, expanded disability status scale; MS, multiple sclerosis; RRMS, relapsing remitting MS; SD, standard deviation; SPMS, secondary progressive MS.

### Increased serum levels of lncRNAs (MALAT1 and lnc-DC) in MS patients

**Figure 1:**
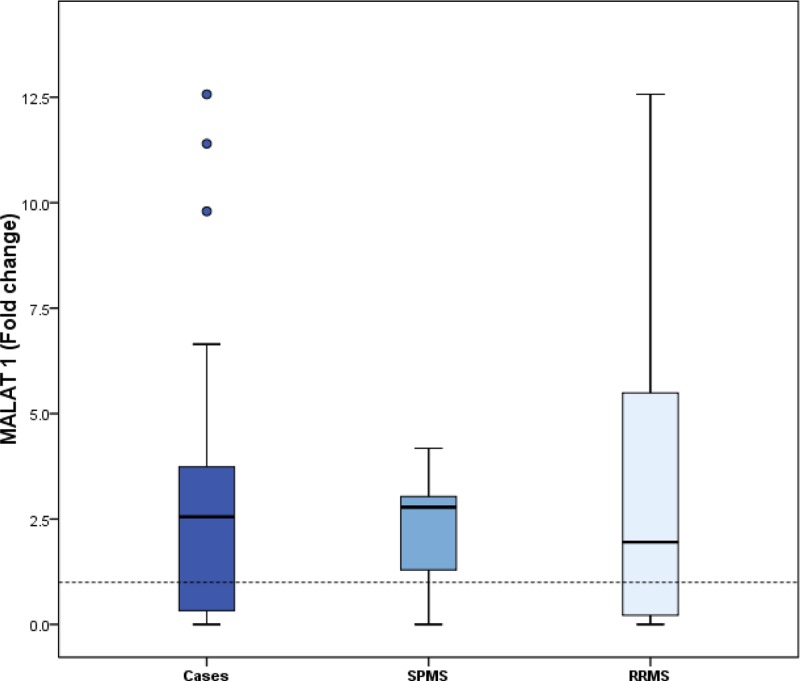
The relative expression of serum level of MALAT1 (fold change) in MS, RRMS and SPMS patients in comparison with normal healthy controls Data are represented by box plot (median, upper and lower quartiles). The horizontal dotted line represents the expression level of normal group. The results showed that the fold change of serum expression level of MALAT1 in MS patients was significantly higher than that in normal subjects, *P*=0.004. The expression of MALAT1 in MS subgroups showed significant elevation of the relative expression of fold change of serum MALAT1 in SPMS patients compared with normal subjects (*P*<0.0001), and no significant difference between RRMS patients relative to normal subjects (*P*=0.119).

**Figure 2:**
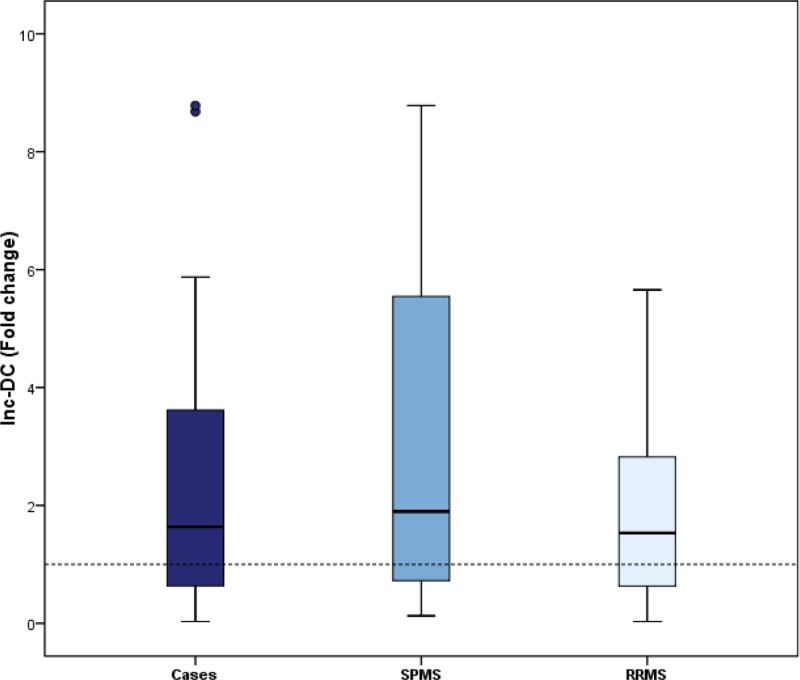
Box plot representation of relative expression level of lnc-DC in MS, RRMS and SPMS patients compared with normal subjects Significant elevation of the fold change of serum expression level of lnc-DC in MS patients compared with normal subjects, *P*=0.006. Relative expression of lnc-DC in MS subgroups and healthy controls showed significant elevation of fold change of lnc-DC in RRMS patients compared with normal subjects (*P*=0.003), and no significant difference between SPMS patients relative to normal subjects (*P***=**0.085). Expression level of the healthy group (equivalent to 1) is represented by the dotted horizontal line.

To determine whether MALAT1 and lnc-DC may contribute to MS or not, the levels of MALAT1 and lnc-DC expression were examined in serum samples from MS patients and healthy control. Figures 1 and 2 showed that fold change of MALAT1 and lnc-DC was both significantly increased in the patients with MS, compared with the normal controls (*P* = 0.004 with 2.55 median fold change and *P* = 0.006 with 1.71 median fold change, respectively). It was suggested that MALAT-1 and lnc-DC might be able to discriminate MS patients from healthy controls.

MS patients were subdivided into RRMS and SPMS subgroups. The present study showed a significant increase in relative expression of serum MALAT1 in SPMS compared with healthy subjects (*P*<0.0001); however, no significant difference was observed as regards the levels of MALAT1 in RRMS relative to healthy controls (*P*=0.119). Concerning the serum level of lnc-DC, it was elevated significantly in RRMS patients (*P*=0.003) compared with healthy subjects. Meanwhile, no significant difference between SPMS subgroup and healthy subjects regarding the level of lnc-DC (*P*=0.085), (Figures 1 and 2) and Table 2.

**Table 2:** Quantitation of serum level (fold change) of MALAT1 and lnc-DC in MS subgroups compared with healthy controls

Variables	MS patients		*P* value^1^		
	RRMS	SPMS	RRMS vs. controls	SPMS vs. controls	RRMS vs. SPMS
**MALAT1** Median (IQR)	1.95 (0.22-5.52)	2.78 (1.28-3.25)	0.119	<0.0001*	0.960
**Inc-DC** Median (IQR)	1.64 (0.63-2.99)	1.89 (0.58-5.71)	**0.003^2^**	0.085	0.310

^1^Mann–Whitney *U-*test, ^2^Significant, IQR, interquartile range; MS, multiple sclerosis; RRMS, relapsing remitting MS; SPMS, secondary progressive MS.

### The association of MALAT1 and lnc-DC expression pattern and disease activity

Disease activity was assessed using EDSS [25]. The results showed no significant correlation between MALAT1 and lnc-DC expression levels and EDSS among MS patients (*r* = 0.105, *P* = 0.491 and *r* = 0.104, *P* = 0.502, respectively).

### Correlations between expression levels of MALAT1 and lnc-DC in MS patients

**Figure 3:**
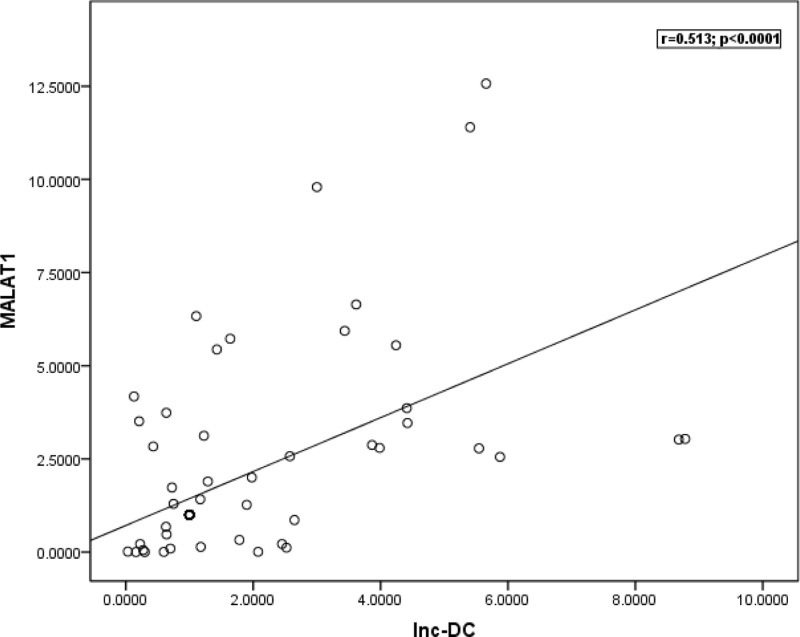
Spearman correlation between the expression levels of MALAT1 and lnc-DC in MS patients. There was a positive association between the expression levels of MALAT1 and lnc-DC (*r* = 0.513, *P*<0.0001) in MS patients.

**Figure 4:**
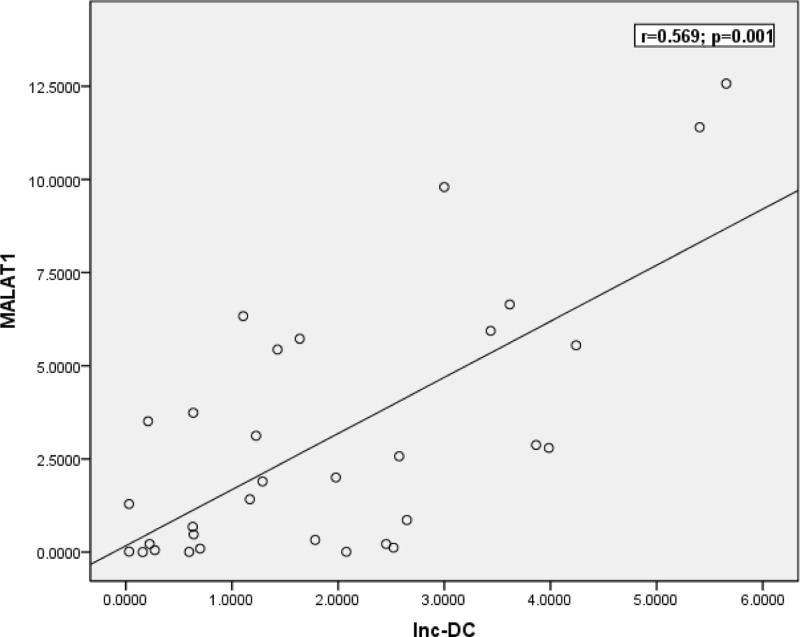
Spearman correlation between the expression levels of MALAT1 and lnc-DC in RRMS Positive correlation was observed between the expression levels of MALAT1 and lnc-DC in RRMS (*r* = 0.569, *P* = 0.001).

According to Spearman correlation analysis, we noted a positive association between the expression levels of MALAT1and lnc-DC (*r* = 0.513, *P* < 0.0001) in MS patients (Figure 3). As regards the association between MALAT1 and lnc-DC expression levels among MS subgroups, positive correlation was observed between them in RRMS (*r* = 0.569, *P* = 0.001) (Figure 4).

### Predictive power of MALAT1 and lnc-DC in MS diagnosis

**Figure 5:**
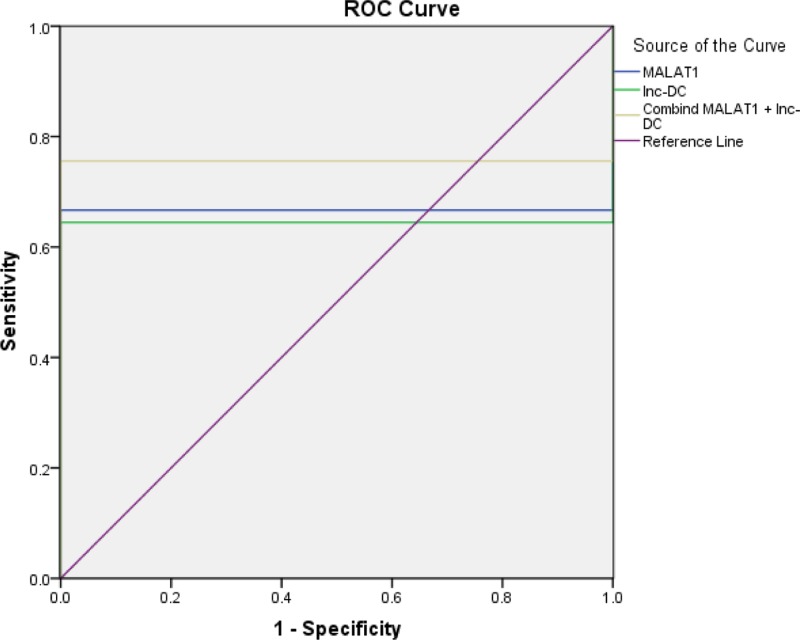
ROC curve for detecting MS using MALAT1 and lnc-DC The AUC value for detecting MS using MALAT1 is 0.667 with sensitivity 66.7% and specificity 100%, and the AUC value for detecting MS using lnc-DC is 0.644 with sensitivity 64.4% and specificity 100%. So, these two parameters can be used for detecting MS, with regards to the AUC of combined MALAT1 and lnc-DC that was 0.756 with sensitivity 75.6% and specificity 100%.

ROC curves analyses were further performed to evaluate the diagnostic value of MALAT1 and lnc-DC through all the MS patients and healthy controls. ROC curve was illustrated in Figure 5 and Table 3. It was observed that both lncRNAs are effective in differentiating MS patients from healthy controls. MALAT1 had an AUC of 0.667 (95% confidence interval (CI): 0.529–0.804*;* sensitivity = 66.7%, specificity = 100% and total accuracy = 83.3), and lnc-DC had an AUC of 0.644 (95% CI: 0.505–0.784; sensitivity = 64.4%, specificity = 100% and total accuracy = 82.2).

**Table 3:** Presentation of MALAT1and lnc-DC in the differential diagnosis MS patients from healthy controls

Variable	AUC 95% CI	Cut-off point	Sensitivity	Specificity	Total accuracy
Malat1 Folds	0.667 (0.529–0.804)	1.134	66.7	100.0	83.3
Inc-DC Folds	0.644 (0.505–0.784)	1.053	64.4	100.0	82.2
Combined	0.756 (0.630–0.881)	0.376 (probability)	75.6	100.0	88.7

AUC, area under curve; CI, confidence interval.

These results indicated that MALAT1 and lnc-DC may be promising biomarkers for MS diagnosis. To identify whether the combination of MALAT1 and lnc-DC could provide better diagnostic accuracy, a binary logistic regression was performed, the result showed that the AUC of combination of MALAT1 and lnc-DC was 0.756 (95% CI: 0.630–0.881, with sensitivity 75.6% and specificity 100% and total accuracy = 88.7), this AUC was considerably higher than the AUC of MALAT1 or lnc-DC separately.

### MALAT1- and Inc-DC-based MS prediction model

**Figure 6:**
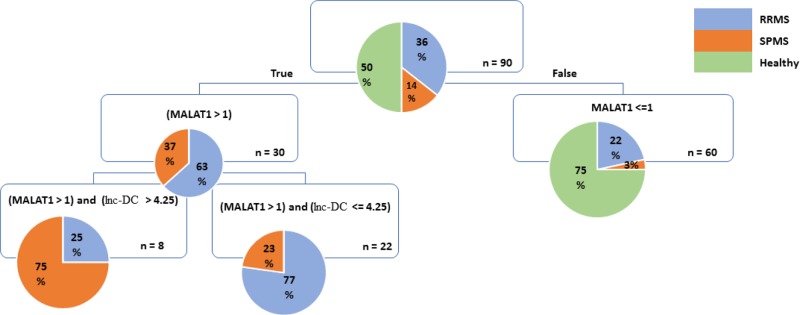
Decision tree model for the prediction of MS patients with differentiation between RRMS and SPMS subtypes The boxes refer to the factors used for decision making.

We investigated the validity of decision tree model for the prediction of MS with differentiation of RRMS and SPMS. The developed decision tree (Figure 6) shows overall propensity mismatch of 24% (i.e. overall performance of 76%) for prediction of MS. As shown, MALAT1 plays the main role for predicting MS, and Inc-DC significantly predicts the type of MS (RRMS and SPMS). The decision tree revealed that if MALAT1 less than or equal to 1, then it is 75% likely of ‘**No** Multiple Sclerosis’. For the high levels of MALAT1, if Inc-DC less than or equal 4.25, then it is 77% likely of RRMS. For the high levels of MALAT1 and Inc-DC, then it is 75% likely of SPMS.

## Discussion

MS is a multicomponent disease causing inflammation, progressive axon loss, formation of demyelinating lesions, and failure of repair mechanisms in CNS, all these changes lead to severe neurological disability. While studying pathogenesis of MS, studies found that the immune dysregulation that involves ‘crosstalk’ between the innate and adaptive immune systems is playing an important role in this disease [26,27].

Many lncRNAs were transcribed in the mammalian genome, and only a small part of lncRNAs have been functionally characterized [28]. Dysregulation of lncRNAs is implicated in the pathogenesis of many neurological disorders as developmental, degenerative and immunological disorders [29,30].

Few studies showed that dysregulated expression of lncRNAs profiles within CNS lesions has a big role in pathogenesis of MS [4,9,31]. There is an interest in studying lncRNAs biomarkers to predict their role in disease activity, progression and treatment response.

Hence, to explore how lncRNAs are implicated in MS pathogenesis and to assess the potential of lncRNAs as predictive biomarkers in MS detection, we demonstrated for the first time the association of two new found lncRNAs (MALAT1 and lnc-DC) among MS patients and healthy controls in our study. We found that MALAT1 expression was significantly elevated in MS patients in comparison with healthy controls (*P*=0.004).

Yang et al*.* [32] studied the role of MALAT1 in the pathogenesis and development of SLE. They detected that overexpressed MALAT1 could induce the expression of SIRT1 and IL-21 in monocytes of SLE patients. It was found that SIRT1 signaling pathway has a role in apoptosis in liver fibrosis [33]. Tegla et al. observed that the expression of SIRT1 was significantly increased in both acute and chronic active lesions in MS brains and peripheral blood mononuclear cells when obtained from patients with RRMS [34]. Several studies reported the role of IL-21 in immunopathogenesis contributing to MS [35,36].

Previous studies revealed that MALAT1 may have a role in the development of retinal neurodegeneration through Cyclic AMP response element-binding protein (CREB) signaling [37]. CREB, a transcription factor, was identified by mass spectrometry analysis as MALAT1-interacting protein and found to be involved in the maintenance of long-term memory. CREB target genes can control the development, function and plasticity of nervous system [38]. The role of MALAT1 was studied in a mouse model of Parkinson disease where it was associated with apoptosis of dopaminergic neurons of the disease [39]. Interestingly, MALAT1 is mainly expressed in neurons, and it has regulated gene expression involved in synapse formation and maintenance in the CNS [17,40]. All previous data imply different possible roles for MALAT1 in the pathogenesis of MS.

In the present study, we also detected higher expression of lnc-DC in MS patients compared with healthy controls (*P*=0.006). Immune abnormalities that occur in MS start with DCs, antigen-presenting cells, which become activated in individuals with MS. These activated cells migrate across the blood–brain barrier and stimulate differentiation of memory T cells into proinflammatory T helper 1 (Th1) and Th17 lymphocytes. In addition, macrophages and microglial cells were activated and produced other proinflammatory cytokines, oxygen and nitric oxide radicals. These substances were responsible for the demyelination and axonal loss [27].

Antigen binding to the cell surface activates DCs, and when communicate with naive CD4+ T cells it activates the adaptive immune response. This process occurs through the immunological synapse and through the production of cytokines by DCs and lymphocytes [41].

Lnc-DC was first expressed in human conventional DCs, and it was found to play an important role in DCs differentiation. Studies proved that knockdown of lnc-DC in human monocytes *in vitro* and in mouse bone marrow cells *in vivo* lead to impaired DCs differentiation and reduced capacity of DCs to stimulate T-cell activation. Lnc-DC bound directly to STAT3 in the cytoplasm, which promoted STAT3 phosphorylation on tyrosine-705, this lead to activation of transcription factor STAT3 and mediated previous effects [19].

Our results are in accordance with a recent study and found that the overexpression of lnc-DC could induce the overmaturation of decidual dendritic cells through the p-STAT3 pathway in pre-eclampsia patients and leads to an increase in Th1 cells [42].

Correlation analysis between MALAT1 and lnc-DC expression levels and clinical data of MS patients revealed no significant correlation between MALAT1 and lnc-DC expression levels and disease activity that was quantitated using the EDSS score (*r* = 0.105, *P* = 0.491 and *r* = 0.104, *P* = 0.502 respectively).

With regards to the correlation between the expression levels of MALAT1 and lnc-DC in MS patients, we observed positive association between the expression levels of MALAT1 and lnc-DC (*r* = 0.513, *P* < 0.0001) in MS patients. In addition, our results demonstrated positive correlation between MALAT1 and lnc-DC expression levels in RRMS (*r* = 0.569, *P* = 0.001). These correlations imply that these lncRNAs might participate in a complex interaction network that regulates expression of several genes with possible role in MS pathogenesis, but further studies are needed to explain these correlations.

Furthermore, ROC curves were constructed for differentiating MS from healthy controls. The results implied that MALAT1 is a more effective biomarker than lnc-DC, with AUC value of 0.667 (95% CI: 0.529–0.804; sensitivity = 66.7%, specificity = 100% and total accuracy = 83.3); however, AUC value of lnc-DC was 0.644 (95% CI: 0.505–0.784; sensitivity = 64.4%, specificity = 100% and total accuracy = 82.2). Combination of MALAT1 and lnc-DC from the logistic regression model demonstrated higher AUC (0.756) when using both markers together than when using each one separately.

Collectively, our study provides evidence that serum levels of MALAT1 and lnc-DC may have great clinical value as accurately promising candidate biomarkers in MS preliminary screening, these lncRNAs may play a role in the pathogenesis of MS disease. In addition, MALAT1 and lnc-DC represent a promising therapeutic target in MS.

Certain limitations in our study are as follows: (i) the population of enrolled patients and controls was relatively small, which needs a larger sample to further study to verify our results; (ii) in terms of ethnicity, further studies are needed to be conducted in different ethnic groups; (iii) more investigations are required to determine the exact molecular mechanisms by which MALAT1 and lnc-DC participate in MS pathophysiology. Whole blood measurement of MALAT1 and lnc-DC in MS is recommended to be done in further works.

## Abbreviations

CNS: central nervous system
CREB: cyclic AMP response element-binding protein
DC: dendritic cell
EDSS: expanded disability status scale
IQR: interquartile range
lncRNA: long non-coding RNA
MALAT1: metastasis-associated lung adenocarcinoma transcript 1
MS: multiple sclerosis
ROC: receiver operating characteristic
RRMS: relapsing remitting MS
SLE: systemic lupus erythematous
SPMS: secondary progressive MS
